# Cardiac diffusion tensor imaging: helix angle (HA) healthy statistical average technique for HA quantification in vivo

**DOI:** 10.1186/1532-429X-15-S1-W7

**Published:** 2013-01-30

**Authors:** PF Ferreira, S Nielles-Vallespin, PD Gatehouse, R de Silva, J Keegan, P Speier, T Feiweier, TG Reese, TF Ismail, A Scott, C Mekkaoui, DE Sosnovik, D Firmin

**Affiliations:** 1 Cardiovascular Biomedical Research Unit, Royal Brompton Hospital, London, UK; 2 National Heart Lung and Blood Institute, National Institutes of Health , Bethesda, MD, USA; 3 Martinos Center for Biomedical Imaging, Massachusetts General Hospital, Charlestown, MA, USA; 4 MR Application & Workflow Development, Siemens AG Healthcare Sector, Erlangen, Germany

## Background

In vivo Cardiac Diffusion Tensor Imaging (cDTI) has great potential to depict the microstructure of the myocardium, with Mean Diffusivity (MD), Fractional Anisotropy (FA), and Helix Angle (HA) maps. Quantitative analysis of these parameters might help disease diagnosis, however HA is particularly difficult to quantify as it varies across the myocardial wall. To overcome this, we developed a method to quantify HA deviation from an average healthy map. This technique, together with quantitative FA and MD analysis, have been applied to healthy volunteer data by varying the number of averages to analyse the improvement of the measurements.

## Methods

The cDTI data of 10 healthy volunteers scanned at 3T during multiple breath-holds at end-systole with a diffusion-weighted STEAM single-shot EPI sequence was used to generate a healthy statistical average LV HA (HSA_HA) map of basal, mid and apical slices (b-value=350s/mm2, 8 averages). Data was post-processed to create FA, MD and HA maps. The overall average range of helix-angles was then used to generate a HSA_HA map for each of the volunteers myocardial shape assuming an epi to endo linear slope of helix-angles as illustrated in higher spatial-resolution ex vivo human DTI scans. These generated HSA_HA maps were used as reference maps to quantify deviation of fibre orientation, assessing the quality of the HA maps measured as the number of averages was increased from 1 to 8.

## Results

**Figure 1:**
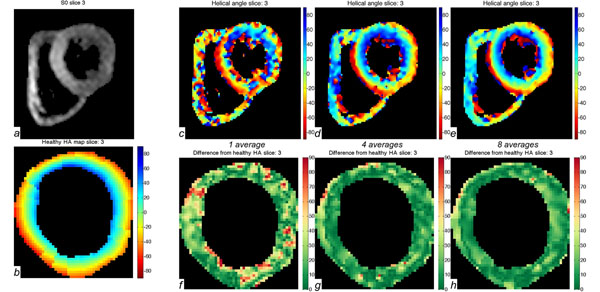
Examples from a basal slice of a volunteer. a: magnitude image. b: HSA_HA (myocardium only) (deg). c-e: HA maps when using 1, 4 and 8 averages respectively (deg). f-h: difference in degrees from the HSA_HA map for 1, 4 and 8 averages respectively (myocardium only).

**Figure 2:**
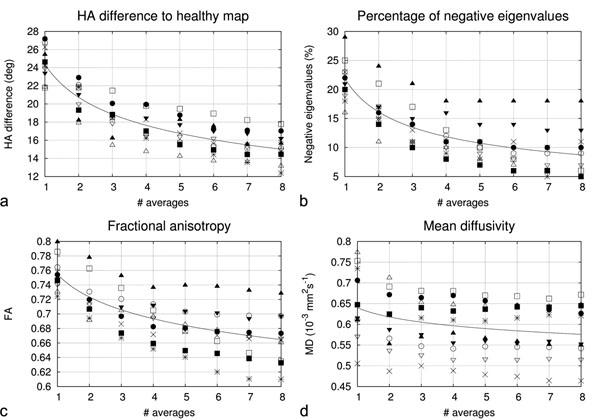
a: Myocardial average difference to HSA_HA map. b: percentage of negative eigenvalues. c-d: FA and MD average for all volunteers as a function of number of averages used respectively. The curve fits are calculated by: F(x)=ax^b.

Figure 1a shows an example of a basal slice of one volunteer. Figure 1b shows the respective myocardial synthesised HSA_HA map. Figure 1c-e illustrates the calculated HA maps with 1, 4 and 8 averages respectively. The pixel-by-pixel difference of HA from the HSA_HA is shown in figures 1f-h. Figure 2 shows the average difference from the HSA_HA map, the percentage of negative eigenvalues, the FA, and MD plotted as a function of the number of averages for all volunteers.

## Conclusions

Synthesising a HSA_HA map from a dataset of 10 healthy volunteers, and matching the myocardial shape of the cDTI data being analysed, allows for a direct pixel-by-pixel comparison of the cardiac fibres’ orientation in vivo. The in vivo nature of the method presented here tolerates comparisons at matching stages of the heart cycle, and will be useful for in vivo quantification of changes in disease hearts, such as periinfarcted regions and cardiomyopathies.

In this study the technique has been applied to determine the quality of the HA maps as a function of the number of averages acquired, showing an asymptotic increase in the quality of data, with changes in HA (along with FA, and MD) lower than 5% per increased average after 5 averages, although this number may increase in patients with more difficulty in breath-holding.

## Funding

This work was supported by the NIHR Cardiovascular Biomedical Research Unit of Royal Brompton and Harefield NHS Foundation Trust and Imperial College London UK, and by the National Institutes of Health (grant: R01HL093038).

